# The patient’s perspectives of safe and routine proactive deprescribing in primary care for older people living with polypharmacy: a qualitative study

**DOI:** 10.1186/s12877-024-05435-x

**Published:** 2024-10-16

**Authors:** D. A. Okeowo, B. Fylan, S. T. R. Zaidi, D. P. Alldred

**Affiliations:** 1 School of Healthcare, University of Leeds, Leeds, UK; 2 School of Pharmacy and Medical Sciences, University of Bradford, Bradford, UK; 3 School of Pharmacy, Newcastle University, Newcastle, UK; 4 NIHR Yorkshire and Humber Patient Safety Research Collaboration, Bradford, UK

**Keywords:** Deprescribing, Implementation, NPT, Primary care

## Abstract

**Background:**

The process of identifying and discontinuing medicines in instances in which harms outweigh benefits (deprescribing) can mitigate the negative consequences of problematic polypharmacy. This process should be conducted with a focus on the patient and involve collaborative decision-making. Evidence is needed regarding patients’ views on how deprescribing should be safely and routinely implemented in English primary care to improve its application. This study aimed to identify optimal methods of introducing and actioning deprescribing from the patient’s perspective.

**Methods:**

Participants in England aged 65 and above who were taking five or more medicines and residing in their own homes were recruited through social media and service user groups. An interview guide was created from deprescribing literature and input from patients and the public, guided by the Normalisation Process Theory (NPT). The interviews were held online using Microsoft Teams^®^ or via phone, recorded, and then transcribed. The data was analysed using the Framework analysis.

**Results:**

Twenty patients (mean age of 74.5, SD = 6.93), with 75% being female, were enrolled in the study. Three main themes emerged: (1) ‘Why deprescribe now?’ emphasised the significance of explaining the reasons behind deprescribing; (2) ‘Monitoring and follow-up’ underscored the necessity of safety measures during deprescribing and patients’ willingness to self-monitor post-intervention; (3) ‘Roles and relationships’ explored patient perceptions of various healthcare professionals involved in deprescribing and the essential interpersonal skills for fostering therapeutic relationships.

**Conclusion:**

Optimal methods of introducing deprescribing included communicating a convincing rationale for stopping medicines and preparing patients for deprescribing conversations. Patients required support from a range of healthcare professionals with whom they had an existing therapeutic relationship. Whilst patients were motivated to self-monitor unwanted/unexpected effects post-deprescribing, timely support was required. The nature of such bolstered collective action and cognitive participation within NPT enhances the normalisation potential of deprescribing. These findings highlight the significance of considering the content and process of deprescribing consultations to enhance normalisation and tackle problematic polypharmacy. This provides a deeper understanding of patients’ needs for implementing safe and routine deprescribing in primary care, which should be considered when designing medication review and deprescribing services.

**Supplementary Information:**

The online version contains supplementary material available at 10.1186/s12877-024-05435-x.

## Introduction

Problematic polypharmacy, where multiple medicines are prescribed inappropriately or the intended benefits are not realised, is a widespread concern [1]. This phenomenon is characterised by the utilisation of potentially inappropriate medicines (PIMs), occurrences of adverse drug reactions (ADRs), adverse drug events (ADEs), and higher care costs [2, 3]. Problematic polypharmacy is particularly a concern for older adults who suffer from a higher burden of medicines, in which PIMs are highly prevalent, and are at higher risk of ADEs and ADRs [4–6]. In conjunction with the natural physiological decline due to ageing, older adults taking ≥ 5 medicines are at an increased risk of problematic polypharmacy [7]. Consequently, there has been a global emphasis on implementing interventions to mitigate problematic polypharmacy, notably through deprescribing [8].

Deprescribing can be defined as the systematic process of identifying and discontinuing medicines when harms outweigh benefits within the context of an individual patient’s care goals, level of functioning, life expectancy, values, and preferences [9]. Empirical evidence has demonstrated that medicines belonging to specific drug classes, such as antihypertensives, can be safely stopped among patients in primary care settings [10]. Furthermore, evidence has highlighted the safe nature of deprescribing interventions, although there are potential risks associated with deprescribing, including symptom relapse and adverse drug withdrawal events (ADWEs) [11]. Research endeavours have recently focused on the proactive nature of deprescribing [12]. Proactive deprescribing seeks to identify and discontinue PIMs pre-emptively to prevent ADRs and ADEs. In contrast, reactive deprescribing entails clinicians responding to explicit clinical cues or situational prompts to stop medicines, often in response to observed ADRs and ADEs [12].

Current evidence about the implementation and safety of routine and proactive deprescribing in primary care settings is lacking [13]. Although previous literature has explored patient barriers and facilitators to the deprescribing process, evidence about community-dwelling older adult patients’ perspectives of deprescribing implementation, underpinned by implementation theory, is needed to support the uptake and sustainability of deprescribing interventions within the UK primary care context [14, 15]. Such empirical evidence is crucial given the heightened susceptibility of older adults to ADRs and ADEs stemming from polypharmacy [16]. Furthermore, applying implementation theory offers enriched comprehension of factors influencing the success or failure of deprescribing implementation [17].

Normalisation Process Theory (NPT) is an implementation framework identifying factors influencing the success or failure of interventions. Comprising four constructs—coherence (how individuals make sense of an intervention), cognitive participation (how stakeholders engage with an intervention), collective action (the work needed for the intervention to occur), and reflexive monitoring (the ongoing evaluation and appraisal of the intervention)—each with four sub-constructs, NPT provides a comprehensive insight into intervention implementation dynamics [18, 19]. As a result, NPT can provide theoretical context to how interventions can be normalised in everyday practice and has been used in such capacity within primary care healthcare research [20].

The aim was to investigate the perspectives of community-dwelling older adult patients concerning the implementation of safe and routine proactive deprescribing in primary care settings. Employing qualitative methods, this study was underpinned by NPT to provide a theoretical understanding of the underlying mechanisms influencing the success of deprescribing implementation.

## Methods

### Study design

This research adopted a qualitative approach with semi-structured interviews conducted with patients. National Health Service (NHS) Ethical approval was received from the East of Scotland Research Ethics Service on 29th March 2021 (ref no. 21/ES/0020). The study was reported in adherence to the Consolidated Criteria for Reporting Qualitative Research (COREQ) [21].

### Recruitment and participants

Purposive sampling was used to identify patients who met the inclusion criteria in Table 1 [22]. The target sample size was 15 to 25 participants, commensurate with previous qualitative deprescribing literature, wherein similar cohort sizes yielded rich data [23–25]. A target sample approach was adopted to allow for a pragmatic approach to managing available study resources. Patients aged ≥ 65 years old taking ≥ 5 medicines were the focus due to their increased risk of problematic polypharmacy [7]. Community-dwelling patients were recruited, and patients living in care homes and receiving palliative care were excluded as such a speciality of care fell outside the remit of this study and require different medicines management processes.

**Table 1:** Participant inclusion and exclusion criteria

**Inclusion criteria**
Patients living in England
≥ 65 years old
Taking ≥ 5 medicines
Community-dwelling
Capacity to provide consent
**Exclusion criteria**
Patients living in care homes
Patients whose care is currently managed through palliative care

Recruitment posters and a participant information sheet were disseminated to service user groups via email and research active GPs through the National Institute for Health Research (NIHR) Clinical Research Network. The study was also shared on the NIHR People in Research website (https://www.peopleinresearch.org/). Informed consent to participate was obtained from all participants in the study. On completion, patients were provided with a £20 Amazon voucher in line with the NIHR INVOLVE guidelines [26]. Recruitment continued until the research team judged a sufficient sample size was achieved based on available resources, remaining timelines, and the volume and quality of data collected.

### Data collection

Semi-structured interviews were up to 80 min in duration and conducted by DO online via MS Teams^®^ or telephone and audio recorded. The research team agreed a maximum interview time limit to allow sufficient time to explore interview questions and avoid unnecessarily burdensome interviews. Participants could have a non-participant with them to provide technology support where necessary. DO also made field notes during interviews to note relevant observations and allow personal reflections on the process.

An interview guide was developed based on the findings of previous scoping and systematic reviews conducted by the research team (Supplementary 1) [15, 27]. It was further refined through team discussions and Patient and Public Involvement and Engagement (PPIE) representatives. PPIE is an approach to research ensuring research is carried out “with” and “by” patients and members of the public instead of “to”, “about”, or “for” them [28]. A hypothetical deprescribing scenario was presented to patients, with subsequent questions based on patient education, patient support, and the potential role of pharmacists in deprescribing in primary care. NPT influenced the development of questions concerning cognitive participation (legitimation) and collective action (relational integration). These questions were around patient involvement and confidence in deprescribing and were included to understand factors needed to normalise deprescribing in primary care. The interview guide was piloted with researchers and PPIE representatives before its use.

### Data analysis

An approved professional transcription company transcribed interviews, and transcripts were not returned to participants for review. Interview data were analysed using framework analysis facilitated by NVIVO^®^ [29]. DO immersed himself in the data, reviewing initial interview recordings and reading transcripts. Reflective field notes were also considered, documenting observations during data collection for additional insights. Open coding followed, with relevant codes assigned to the data. These codes were refined and grouped based on characteristics and similarities. The iterative process resulted in an analytical framework comprising distinct code categories. Finally, the established framework was applied to the remaining transcripts, with the researcher critically reviewing codes and categories concerning the research question. The coding process was conducted by DO, with BF, TZ, and DPA providing feedback on the coding framework. NPT was used in this process to guide interpretation during the framework process, particularly of the NPT-derived questions, and to contextualise findings.

## Results

### Participant characteristics

Twenty participants were recruited, with 10 online and telephone interviews each conducted. No repeat interviews were conducted, and no participants dropped out. The mean participant age was 74.5 (SD = 6.93), with most participants within the 71–80 age range, and 75% of participants were female. Most of the study population consisted of female participants, and the most common ethnicity was white British. One participant (participant 20) attended the interview with a non-participant (family member) who consented to the study but did not contribute. The interview duration ranged from 23 to 71 min (mean 46.75 min). Findings were disseminated to participants to provide the opportunity for feedback. However, no changes were made to the primary findings produced. The research team agreed that the data were sufficient after conducting 20 interviews.

### Findings from Framework Analysis

**Fig. 1:**
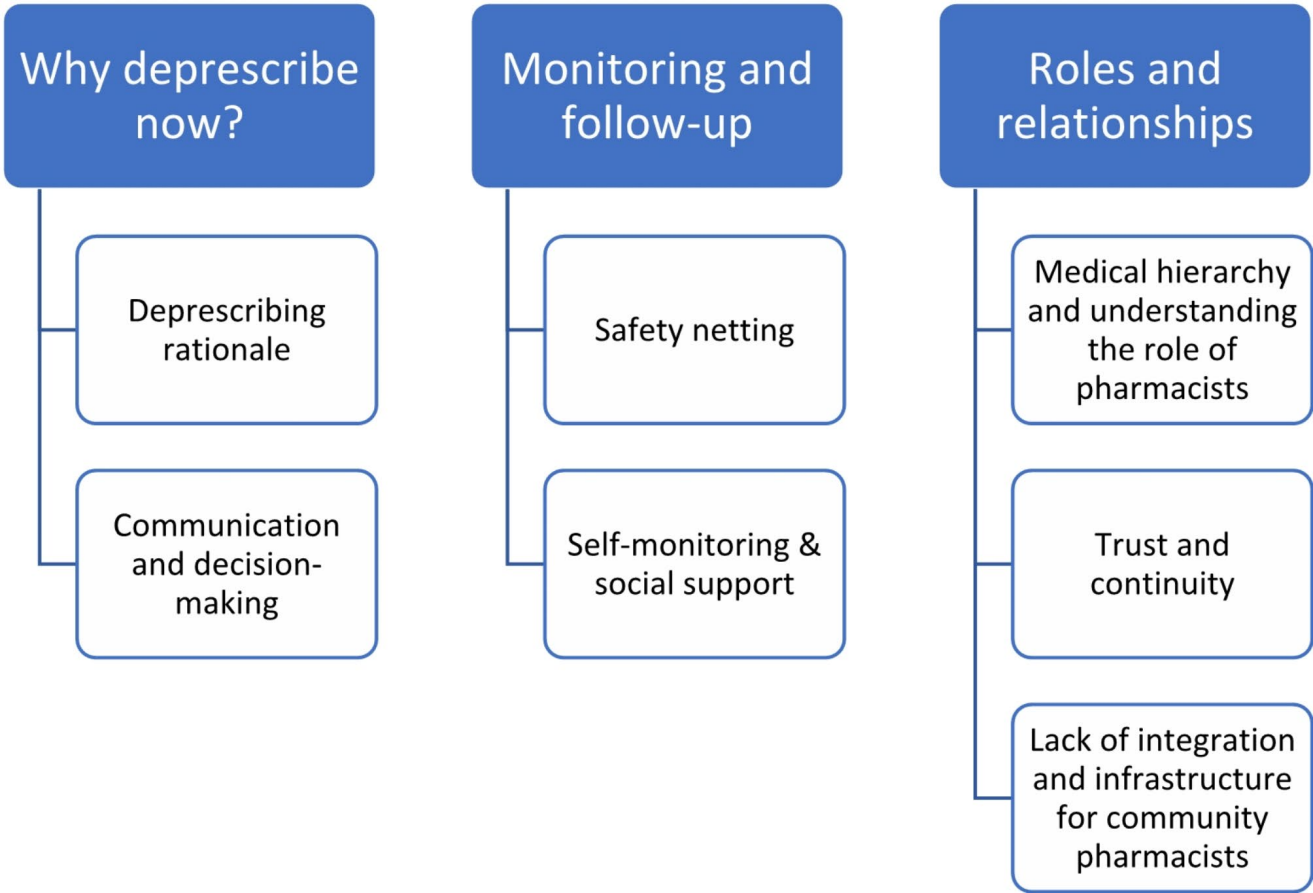
Thematic map derived from Framework analysis of participant interviews

Following framework analysis, three themes were developed, with eight associated subthemes (Fig. 1). These main themes were: ‘Why deprescribe now?’, ‘Monitoring and follow-up’, and ‘Roles and relationships’.

### Theme 1: Why deprescribe now?

This theme describes information patients would like when deprescribing is introduced.

#### Subtheme 1: Deprescribing Rationale

When a deprescribing recommendation was proposed, participants were primarily concerned about the rationale supporting the recommendation. Participants explained they would question what may have changed in their health to trigger healthcare professionals (HCPs) to consider stopping a medicine. This led to participants considering what harm would occur if they continued the medicine instead.

*“I would always ask the question why* [is it being proposed that a medicine should be stopped], *why now*,* why this*,* what do you expect would happen?”* [Participant 9, female, 79].

Participants expressed why understanding the rationale for deprescribing was critical to accepting deprescribing. They had previously been told their medicines were ‘for life’, and the idea of medicines being stopped contradicted this, with patients taken aback by, or sceptical of, deprescribing. Similarly, participants explained how being provided with, and sufficiently convinced by, the rationale for deprescribing enhanced their confidence in deprescribing.

*“The thing that would make me feel most confident* [in deprescribing], *if I was given good convincing advice as to the reason for stopping that medication”* [Participant 1, male, 88].

It was essential for participants to be reassured that deprescribing was not financially motivated. Numerous participants disclosed that they would be suspicious that deprescribing was predominantly due to the NHS needing to save money. This was due to their experiences with medicine changes due to cost savings, such as medicine brand switches.

*“I’m assuming that simply stopping*,* and this is being a bit cynical*,* is for my benefit and not the budget’s benefit.”* [Participant 17, female, 74].

Subsequently, participants wanted to know about the deprescribing plan i.e., whether this would be a gradual dose reduction or an abrupt stop, whether another medicine would need to be introduced as an alternative, and the potential side effects or benefits they might experience. Participants clarified that this further information would help them decide whether deprescribing was appropriate.

*“Probably timelines. Was it going to be something that it would just stop*,* bang? Is it something that’s gradually phasing out?”* [Participants 14, male, 66].

A minority of participants expressed interest in the evidence supporting deprescribing. They would be inclined to participate in deprescribing if research was provided to explain why it would benefit them specifically. These participants stated they were motivated by evidence and emphasised deprescribing evidence as part of the explanation. Furthermore, support for deprescribing from healthcare governing bodies would help substantiate the reasoning.

*“If there is some research* [about stopping a medicine], *I’d want to know the source of that.”* [Participant 7, female, 83].

Participants noted that learning about other patients’ experiences with deprescribing would help improve their confidence in the process. This could be through word of mouth or reading about other patients’ deprescribing experiences. Knowing that other patients had been through a similar situation safely was described as motivating, relatable and provided confidence that patients would not be alone in their deprescribing journey.

*“Other people’s experience… Because of other people’s experience taking a lot of medication*,* like myself*,* and having to reduce it slowly*,* it makes me feel better that I have something I can relate to… Confidence in yourself that you are not alone.”* [Participant 18, female, 72].

#### Subtheme 2: Communication and decision-making

Participants preferred verbal deprescribing conversations, either face-to-face or over the phone, supplemented with written information about what was discussed post-consultation. Participants also wanted to be directed to additional information sources, such as websites, that they could access. It was emphasised that such material would assist participants in remembering what had been said, explaining the conversations to carers or family members, and aiding their decision-making following the consultation.

*“I’d like it face-to-face and at the same time to be given a leaflet as*,* when you’re with somebody*,* you don’t always take all the facts onboard. So*,* to have face-to-face and then to be given a written explanation.”* [Participant 10, female, 87].

Supplementary written material to aid decision-making was consistent with participants’ wishes to be appropriately prepared for deprescribing consultations, allowing them to be more involved in these conversations. They described how this could be accomplished by providing patients with advance notice of the consultation so that they can prepare their thoughts or by splitting the consultation into two parts, with the first consultation focusing on information sharing and the second consultation being when a decision is made, allowing patients to reflect on the information and prepare for a decision in the second consultation. This also allowed for objective assessments of their health (regarding the medicine to be deprescribed) before reaching a decision in the second meeting.

[How patient imagines doctor would introduce deprescribing] *“I’ll give you the online information… We’ll make an appointment in two weeks’ time and then we can come in and review. Once you’ve had a chance*,* we can talk through it.”* [Participant 11, female, 66].

Participants who valued being well-prepared for discussions about deprescribing generally agreed that they have a role to play in these conversations, with only one exception among the participants interviewed.

*“I should be involved* [in deprescribing discussions] *… I believe I can be now.”* [Participant 10, female, 87].

Participant emphasised the importance of effective communication between patients and HCPs involved in their treatment to ensure that deprescribing recommendations were consistent across HCPs. This meant that if one HCP advised or conducted deprescribing, another HCP would not oppose the decision. Ideally, a decision to deprescribe a medicine would be conveyed to all HCPs actively participating in that patient’s care, and someone would serve as a central coordinator to carry out the deprescription.

*“Just to know who’s coordinating* [deprescribing], *rather than have the doctor say one thing and then a pharmacist saying another… Knowing that they’re communicating with each other.”* [Participant 19, female, 72].

### Theme 2: Monitoring and follow-up

This theme presents the nature of support patients believe they require from HCPs during and after a medicine has been deprescribed.

#### Subtheme 1: Safety netting

Participants voiced how they felt it was necessary for HCPs to follow-up with patients during and after deprescribing to ensure safety. However, there was a lack of consensus on frequency, ranging from weekly to yearly. Patients deemed this was dependent on the medicine being deprescribed, the patient’s current clinical status and results from any clinical tests conducted. Participants were content to delegate this to the HCP as they were best equipped to decide.

*“I would hope that my doctor would say*,* I will see you again in three months’ time or whatever*,* and see how it is going. If you have a blood test just before then*,* we will know where we are and take it from there. And then maybe another three or six months or two weeks or whatever. There would be that kind of plan and aftercare from the decision to stop.”* [Participant 8, female, 67].

Most participants showed no real preference for who provided this support as long as they were qualified and the patient was made aware. Participants expressed how they may not completely understand the roles, responsibilities, and skills of different HCPs so could not dictate who was best qualified to provide deprescribing support.

*“Whoever’s best equipped at that time. There’s very little method of understanding the ability of a doctor*,* or a pharmacist*,* or a nurse*,* we’re not qualified to be judge and jury on all those professions*,* they’re there to advise us and guide us in what’s best for us.”* [Participant 1, male, 88].

Participants appreciated the idea that community pharmacists could serve as a safety net during deprescribing. Pharmacists could contact patients during or after deprescribing to check on their well-being, and patients who became ill after deprescribing could attend the pharmacy. This would give patients a contact aware of the deprescribing and could assist with questions or reassurance. One participant compared this idea to the current NHS New Medicine Service (NMS), a free pharmacy service to help patients maximise the benefit of newly prescribed medicines for long-term conditions [30].

*“I’ll stop the medicine. That’s fine. If I realise a week later that I’m not feeling well or I think this may be due because I’ve stopped the medicine*,* I can walk to my community pharmacy and ask the pharmacist and have a chat.”* [Participant 12, male, 74].

#### Subtheme 2: Self-monitoring and social support

Although follow-up was seen as essential to ensure the safety of deprescribing, participants were cognisant that regularly scheduled follow-ups, especially when patients were well, may be unnecessary and increase patient anxiety. Participants were also aware of constrained clinician time and that unnecessary follow-ups would exacerbate this. As a result, participants emphasised the benefit of empowering patients to self-monitor after deprescribing and then consult with an HCP when needed.

*“We’re not talking about complex medicines*,* dangerous medicines here*,* so there is no need to check up unless the patient says I can’t take it*,* I’m feeling worse… It could be at the next consultation or by emailing the GP surgery. But keep a little journal. Keep a little diary of what happened to your symptoms.”* [Participant 12, male, 74].

Participants stated that to be best equipped to self-monitor, they needed to be aware of potential red flag symptoms, what to do if they occurred, and that the stopped medicine could be restarted if necessary. Participants also wanted a point of contact who, while not necessarily their own GP, was aware of the deprescribing and could answer related questions. This was critical for participants who had expressed concerns about difficulty contacting HCPs during the COVID-19 pandemic and did not want to be in a similar situation during deprescribing.

*“I think having the means of easy contact if you’re concerned about something*,* not going through trying to get an appointment*,* which is a hassle… If I’m in that situation where there’s a possibility of something arising*,* I would like to know who I can turn to immediately?”* [Participant 17, female, 74].

Participants highlighted the potential need for emotional support or counselling as they may be emotionally attached to their medicine, and having to stop taking them may disrupt their lifestyle and cause anxiety. In addition, ongoing or acute stressful situations that HCPs are unaware of may be intensified by deprescribing. Participants also voiced how counselling may help living with their long-term condition, which could be expanded to help patients learn about non-pharmacological treatment options to complement deprescribing, such as coping strategies to deal with pain if analgesics were deprescribed.

*“For example*,* if it is pain relief* [being deprescribed], *then there might be other temporary mechanisms they could teach me*,* like mindfulness or other coping strategies”* [Participant 19, female, 72].

### Theme 3: Roles and relationships

This theme describes patients’ views about the roles and relationships of HCPs in deprescribing.

#### Subtheme 1: Medical hierarchy and understanding the role of pharmacists

Participants often stated that they would only accept deprescribing if their GP explicitly agreed with the recommendation. Furthermore, participants stated they did not want other HCPs, such as GP-practice pharmacists, to deprescribe without consulting their GP. Some participants stated that they would prefer their GP to propose deprescribing, which could then be followed up by another HCP.

*“I would*,* if the doctor advised it*,* be happy for the GP pharmacist to oversee the reduction or stopping of medication*,* but I don’t think I would be happy with them initially instigating the process.”* [Participant 3, female, 76].

Participant valued pharmacists’ involvement in different stages of deprescribing, whether practice- or community-based. Based on their qualifications and training, participants recognised pharmacists as medicines specialists and could recall positive experiences discussing medicine queries with pharmacists. As a result, as deprescribing involves medicine use, participants found it ideal that a pharmacist would be involved in the process.

*“I found that local pharmacists are usually very helpful. You only have to say*,* can I ask you a question and they say yes*,* certainly*,* what is it? … There is usually a pharmacist who will come and listen to your questions and answer them if they can.”* [Participant 8, female, 67].

Participants perceived an advantage to the involvement of GP-practice pharmacists in that there was an assumed close working relationship with their GP, which was deemed beneficial. This emphasised the importance of GP’s involvement. In comparison, the involvement of community pharmacists had the advantage of making community pharmacies more accessible to patients. A participant mentioned that patients could easily contact their community chemist if they needed quick advice on deprescribing.

*“I would say the community pharmacists because it would be easier in terms of practicalities. It would be easier for the patient to go to the community pharmacy.”* [Participant 8, female, 67].

When asked about the potential role of pharmacists in deprescribing, most participants highlighted that they would seek advice from a community- or GP practice-based pharmacist when deciding if deprescribing was right for them. There was an expectation that pharmacists could provide additional advice on the importance of, or reasoning for, deprescribing and any practical changes around medicines management that might need to be considered, such as the supply of medicines post-deprescribing.

*“Well*,* I would expect them* [community pharmacists] *to be able to assist me too*,* with advice on the effects of* [deprescribing], *the effects of diminishing the supply and so on.”* [Participant 1, male, 88].

Pharmacists’ deprescribing advice was also favourable to participants as they valued additional HCPs’ involvement in their care. Participants felt pharmacists could highlight things their GP had missed, enhancing their safety and care. As a result, participants welcomed the opportunity for pharmacist involvement.

*“Yes*,* definitely*, [Pharmacists involvement in deprescribing advice] *means they’re looking after my welfare*,* which would be important. I would hate to think they’d hesitate if they knew something and maybe thought*,* I wonder if the GP knows this* [medicine could be stopped].*”* [Participant 7, female, 83].

One participant proposed accessing deprescribing leaflets in community pharmacies. The leaflets would introduce deprescribing, which could then be followed up with a consultation with the community pharmacist in a consultation room if the patient had more questions.

*“Information sharing first*,* and a leaflet would be fine… Just to introduce the subject at first. And then perhaps a more one-to-one talk in the little office to ask more questions about it.”* [Participant 14, male, 66].

When asked about the prospective responsibilities of community pharmacists, several participants liked the concept of pharmacists finding and conveying deprescribing recommendations to their GP. In this capacity, participants wanted pharmacists to assess the need for their medications to find prospective medicines that could be deprescribed once directed to the patient’s GP. However, participants stated that before the deprescribing recommendations were given to their GP, the pharmacist and the patient needed to discuss them. Furthermore, a second consultation with the GP about the recommendations should take place before deprescribing was commenced.

*“‘Do I need to be taking these?’*,* and they* [community pharmacist] *could perhaps contact the doctor and just say he’s brought this up*,* I think there may be some justification*,* could you see him?”* [Participant 2, male, 66].

In contrast, not every patient supported the idea of pharmacists participating in deprescribing. Some participants stated that they did not fully understand pharmacists’ current roles, referring to how they previously understood pharmacists’ responsibilities to be solely in dispensing. Some participants believed that pharmacists’ new and developing roles would dilute the roles of GPs and that, because pharmacists were viewed as “in the back checking things” [Participant 13], they may lack interpersonal skills to speak with patients due to a lack of exposure. This eroded some participants’ trust in pharmacists’ ability to be involved in deprescribing.

*“I don’t know the role of the pharmacist really. At one time they were there dispensing medicine. And now they seem to have taken the role that once was the GP’s. So I don’t know whether I’d be comfortable with a pharmacist stopping the medicine.”* [Participant 4, female, 70].

#### Subtheme 2: Developed trust and continuity of staff

Participants emphasised the pre-existing developed relationships and trust between patients and HCPs. This developed relationship and trust were pivotal to accepting deprescribing, and when this trust/relationship had previously been perceived as compromised, participants held negative perceptions of the involvement of that HCP. Therefore, in many cases, it was not necessarily important which HCP should be involved in deprescribing based on their profession or skills, but the pre-existing relationship between that HCP and the patient.

*“I think the relationship you’ve got with them must be paramount in* [deprescribing].*”* [Participant 14, male, 66].

Participants expressed concern about not seeing the same HCPs involved in deprescribing and a desire to see the same HCPs to form a therapeutic relationship if one did not already exist. There was a particular concern about continuity with pharmacists, with participants sometimes feeling that the pharmacist “doesn’t know” them because of a lack of history of interaction with them, especially if the pharmacists covered multiple practices or community pharmacies.

*“But in a tiny GP surgery where the pharmacist works for three different GP surgeries in a week*,* that doesn’t really work. So*,* if a GP pharmacist I don’t know stopped a medicine*,* I would need something in writing.”* [Participant 12, male, 74].

There was concern about a potential conflict of interest between practice pharmacists and GPs. One participant believed that because practice pharmacists work for GPs, they would be required to agree to clinical decisions and recommendations made by GPs or risk losing their jobs. This potential conflict of interest would undermine the patient’s trust in deprescribing.

*“Don’t forget [practice pharmacists] also work for the GP surgery. So*,* they have a conflict of interest. If they don’t agree with the prescribing that a GP is doing*,* they can’t do much about that because they find themselves without a job”* [Participant 12, male, 74].

Finally, there was also some concern about the involvement of community pharmacy staff other than the pharmacist. Although satisfied with the involvement of community pharmacists in deprescribing, some participants highlighted they would not want to involve other community pharmacy staff, such as counter assistants. Patients stated they would not want to discuss confidential information with them.

*“As long as it was the pharmacist and not just one of the assistants standing behind the counter who was helping.”* [Participant 10, female, 87].

#### Subtheme 3: Lack of integration and infrastructure for community pharmacists

One of the main concerns raised by participants when considering community pharmacist involvement in deprescribing was a lack of integration with broader primary care. Particularly a lack of access to complete medical records and clinical services, such as ordering and reviewing blood tests. As a result, several participants found it difficult to imagine how community pharmacists could assist with deprescribing, as their involvement would be limited by a lack of knowledge of the patient’s full clinical history, or their clinical actions would be limited by a lack of access to broader primary care services.

*“I think the difficulty we have with that is* [community] *pharmacists can’t order*,* for example*,* a blood review. It would have to go back to the surgery for somebody to authorise blood reviews.”* [Participant 15, male, 76].

The physical environment in which HCPs work was also critical to implementing deprescribing. Participants stated that having a private area to discuss deprescribing would be critical. Patients feared discussing their medical history, particularly regarding deprescribing, over the counter in a community pharmacy, so a consultation room was essential.

*“To have somewhere where the conversations can happen. So*,* at the moment*,* and I said earlier*, [my community pharmacy] *hasn’t got a room*,* so I wouldn’t want it to happen over the counter with other people around.”* [Participant 19, female, 72].

## Discussion

### Key findings

The significance of a robust rationale for deprescribing cannot be overstated. Participants frequently expressed a need to comprehend the reasoning behind deprescribing initiatives, often voicing concerns about potential financial motives and questioning the practicality of such interventions. The association of deprescribing with cost-saving measures, such as switching medicine brands, exacerbated scepticism among patients [31]. However, engaging in discussions concerning the clinical rationale behind deprescribing can ease doubts and foster patient acceptance.

Participants often questioned the feasibility of deprescribing, recalling instances where they were informed that certain medicines would be lifelong commitments. This ingrained notion presents a challenge to HCPs when addressing patient perceptions regarding deprescribing. The language used when initiating prescriptions significantly influences subsequent attitudes toward deprescribing [32]. When patients are consistently advised that a medicine is lifelong, altering this mindset becomes challenging, underscoring the necessity for a compelling rationale when introducing deprescribing.

Participants advocated for sufficient time to evaluate evidence before making decisions regarding deprescribing. A two-stage deprescribing consultation model was proposed, allowing for initial information dissemination and subsequent reflection, bolstering shared decision-making. This aligns with guidelines promoting patient involvement in treatment decisions, emphasising the importance of allowing patients time to make informed choices [33]. However, the increased clinician time required for a two-stage deprescribing consultation must be adequately justified as such an approach may not be feasible with current constrained resources [34].

Within the NPT collective action construct, patient confidence emerged as a crucial factor in facilitating deprescribing in primary care. Patients’ trust in the rationale behind deprescribing was pivotal for collective action, that is, the work needed to make deprescribing occur routinely. Allocating time to ensure patients understand and accept the rationale, process, and potential implications of deprescribing was imperative for instilling confidence and promoting patient engagement. This has identified an avenue in which the normalisation of deprescribing can be enhanced through efforts to provide patients with a sufficient deprescribing rationale.

Participants expressed a readiness to be involved in deprescribing consultations once adequately prepared for them i.e. notice of the consultation. Within the lens of NPT, this enhanced cognitive participation, which is the relationship work that stakeholders in an intervention conduct to normalise the practice. As a result, adequately preparing patients for deprescribing consultation so that they could be subsequently involved in such consultations bolstered the normalisation potential of deprescribing. Through identifying these findings, both constructs of NPT (collective action and cognitive participation) can be augmented to enhance normalisation.

Participants deemed follow-up appointments with HCPs and self-monitoring post-deprescribing essential to mitigate potential adverse outcomes. Incorporating self-management strategies empowers patients and has been shown to enhance health outcomes [35, 36]. Participants envisioned a role for community pharmacists in deprescribing support, similar to that of the NHS NMS [30], but highlighted the importance of communication and continuity of care among healthcare providers.

While some participants endorsed the involvement of community pharmacists in deprescribing, others expressed reservations, citing concerns about deviation from traditional pharmacist roles in dispensing and subsequent dilution of GP responsibilities. This follows a long-standing theme within pharmacy literature, where patients held similar views regarding pharmacists within extended non-dispensing roles and prescribing [37]. Understanding patient perspectives is crucial in navigating the evolving landscape of deprescribing practices within primary care.

### Comparisons with existing literature

Although there is a scarcity of literature exploring the significance of deprescribing rationale, other studies and reviews have examined the barriers and facilitators to deprescribing. Reeve et al. (2013) conducted a systematic review exploring patient-reported barriers and enablers to deprescribing [32]. The review found three key themes which acted as barriers and facilitators to deprescribing. These were the appropriateness of cessation, the process of cessation, and influences. In addition, fear was an additional barrier, whilst a dislike for medicine was an additional facilitator [32]. Notably, the study reinforced how patients judging whether deprescribing is appropriate could act as a barrier or facilitator towards accepting deprescribing [32]. Comparable systematic reviews exploring barriers and facilitators to deprescribing have also identified similar findings, emphasising the importance of addressing patient perception and understanding of deprescribing [15, 38]. This highlights the link between patients’ perceived appropriateness of deprescribing and the significance and nature of the deprescribing rationale presented in this study.

This study extends previous research by identifying that the deprescribing rationale and how it is communicated to patients is pivotal to patient-perceived deprescribing appropriateness and a facilitator of deprescribing implementation. Furthermore, this study provides insight into the significance of the deprescribing rationale to bolster the perception of appropriateness to patients and enhance deprescribing normalisation. For example, the importance of separating deprescribing from cost-saving exercises to enhance patient confidence in the deprescribing rationale, promote collective action within the domain of NPT, and bolster the normalisation potential of deprescribing. Equally, preparing patients to discuss the appropriateness of deprescribing, an enabler to deprescribing highlighted by Reeve et al., (2013), can support patients to be involved in deprescribing consultations [32]. This directly works on the NPT construct cognitive participation and enhances deprescribing normalisation. As such, the findings of this study shed greater light on the barriers and facilitators to deprescribing, identified by Reeve et al., and their effect on implementation. This furthers the science of how deprescribing can be delivered as appropriate and beneficial towards patients, enhancing its implementation in primary care.

With patients envisioning a role for community pharmacists in deprescribing, it is imperative to understand patient perceptions of the role of community pharmacists. Hindi et al., (2017), in their systematic review of patient and public perspectives on community pharmacy services in the UK, reported low public and patient awareness regarding extended pharmacy services, with limited recognition of community pharmacists beyond traditional dispensing roles [39]. Clinical services offered by community pharmacies, including the NMS, were poorly understood and underutilised. Lack of awareness was attributed to a lack of promotion of these services [40]. One study identified resistance to acknowledge pharmacists as essential members of the healthcare team, with patients questioning the appropriateness of the extended roles of community pharmacists, with perceptions of pharmacists “behind the counter” with roles limited to dispensing and minor conditions. Concerns were raised regarding potential commercial affiliations, financial motives, and perceived limitations in knowledge and training beyond dispensing. Despite this reluctance, participants recognised the expertise of pharmacists in medicine-related matters. Features that enhanced the use of pharmacy services included ease of access and convenience, while perceived lack of privacy and confidentiality acted as barriers. The review highlighted a theme of doctor supremacy, with respondents favouring doctor involvement over pharmacist involvement regardless of the service provided [39].

These findings align with the results of this study, particularly patients’ recognition of the benefits of community pharmacy. However, the hierarchy of GPs in deprescribing decision-making and patients’ limited understanding of pharmacy roles beyond medicine supply were also observed, emphasising the need to increase awareness of the suitability of community pharmacists in deprescribing interventions. There is also the need to build therapeutic relationships and trust between community pharmacists and patients to enhance patient utilisation of pharmacy services.

### Strengths and limitations

This study has provided valuable insights into patients’ perspectives on deprescribing implementation in primary care. A key strength of this study was the incorporation of NPT as underpinning theory. By asking questions related to patient confidence and involvement in deprescribing, derived from the sub-constructs of relational integration and legitimation in NPT, a deeper theoretical understanding was gained regarding the factors that support patient confidence and involvement in deprescribing and their significance in the process of normalising deprescribing practices.

However, several limitations are acknowledged. To comply with COVID-19 social distancing restrictions, interviews were conducted online or via telephone. This may have excluded patients who were not comfortable with these methods. Efforts were made to minimise this limitation by assisting participants in connecting online or via telephone for interviews. Reduced access to HCPs during multiple lockdowns may explain why participants emphasised the importance of seeing and/or speaking with HCPs during deprescribing. However, it is well-documented that patients faced challenges in accessing care services, particularly in primary care, before the pandemic [41]. The accessibility of community pharmacists has also been noted in the literature before the pandemic [42]. Another limitation was the lack of ethnic diversity in the study population. Finally, it was observed that some participants recruited through the NIHR People of Research website frequently participate in healthcare research and may possess a higher healthcare knowledge level than the general population. Although evident in some interviews, the number of participants recruited through the NIHR People of Research was less than a quarter of the study population.

### Reflexivity

It is important to consider the researcher’s positioning and reflexivity in qualitative research to understand nuanced subjectivity in the research. The primary researcher (DO) is a male pharmacist conducting this research as part of their doctoral training. As such, it is appreciated that their clinical experience and medicines optimisation knowledge may have influenced data interpretation. However, it is believed that working in a research group with non-healthcare professionals, PPIE representatives, and academics with extensive experience helped balance such influence.

## Conclusion

Participants expressed their preferences regarding the information conveyed during deprescribing discussions, the requisite support for safe deprescribing, and the involvement of pharmacists. They stressed the significance of a comprehensive deprescribing rationale, patient deliberation time, inter-HCP communication, and pharmacist consultations. Patient confidence following a sufficient deprescribing rationale bolstered collective action, whilst adequate time to prepare for such consultations aided patients in deprescribing involvement, thus enhancing cognitive participation in the intervention. As both collective action and cognitive participation enhance the normalisation of interventions, significance should be placed on preparing patients for deprescribing consultations and how the rationale is conveyed. Participants were willing to self-monitor post-deprescribing, contingent upon a designated point of contact. They endorsed multi-HCP involvement in deprescribing, provided there was prior communication, continuity of care, and GP involvement. These insights contribute to advancing the normalisation of routine deprescribing in primary care.

## Electronic supplementary material

Below is the link to the electronic supplementary material.

Supplementary Material 1

## Data Availability

The datasets generated and/or analysed during the current study are not publicly available due to ethical reasons but are available from the corresponding author on reasonable request.

## Abbreviations

ADE: Adverse drug events
ADR: Adverse drug reaction
ADWEs: Adverse drug withdrawal events
COREQ: Consolidated Criteria for Reporting Qualitative Research
GP: General practitioner
HCP: Healthcare Professional
NHS: National Health Service
NIHR: National Institute for Health Research
NMS: New Medicine Service
NPT: Normalisation Process Theory
PIMs: Potentially inappropriate medicines
PPIE: Public Involvement and Engagement
