# Supplementary figures and images for: The patient’s perspectives of safe and routine proactive deprescribing in primary care for older people living with polypharmacy: a qualitative study
Source: BMC Geriatr. 2024 Oct 16;24:844. doi: 10.1186/s12877-024-05435-x (PMC11481278; doi:10.1186/s12877-024-05435-x)

Supplementary file 1: Interview guide
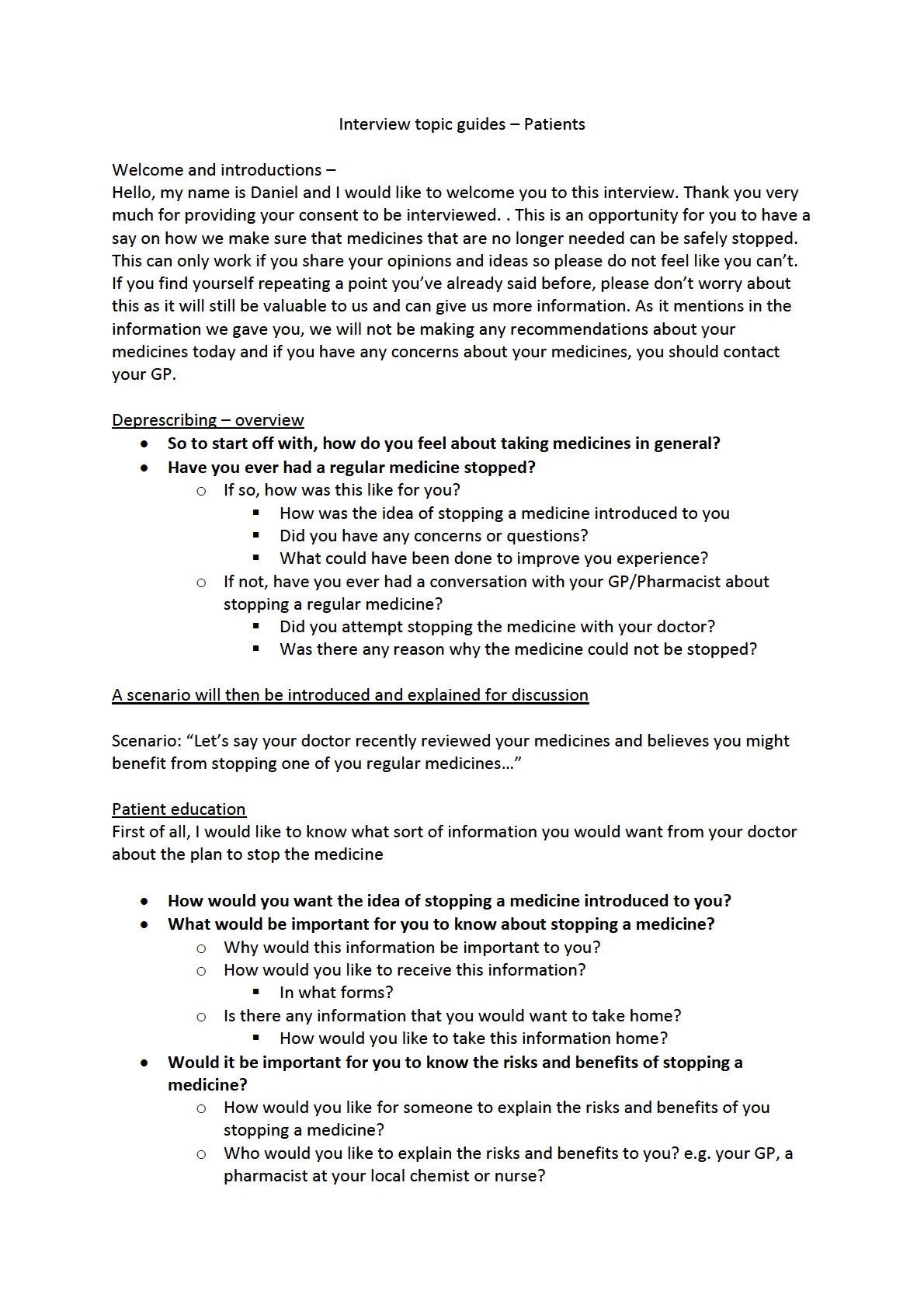


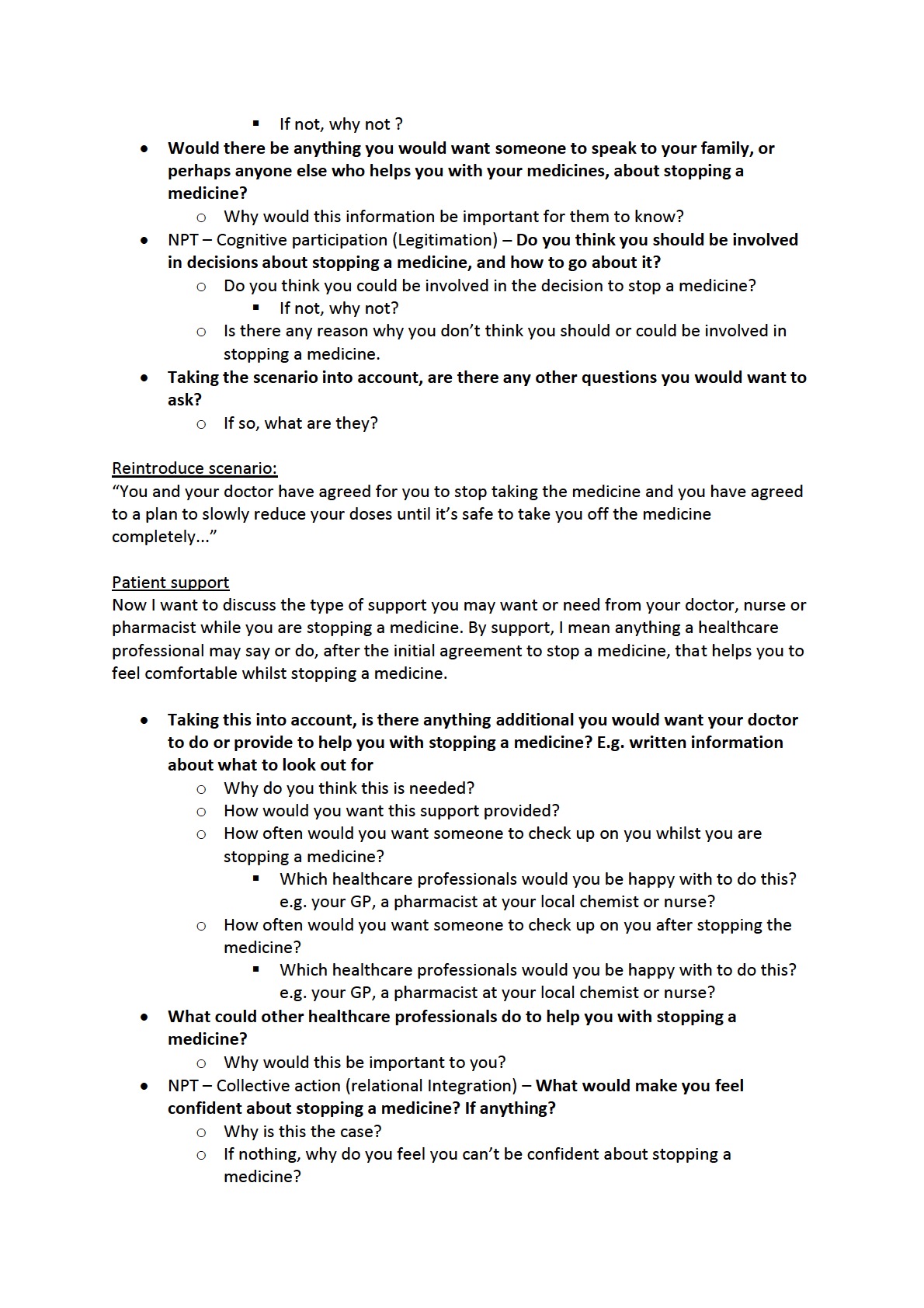

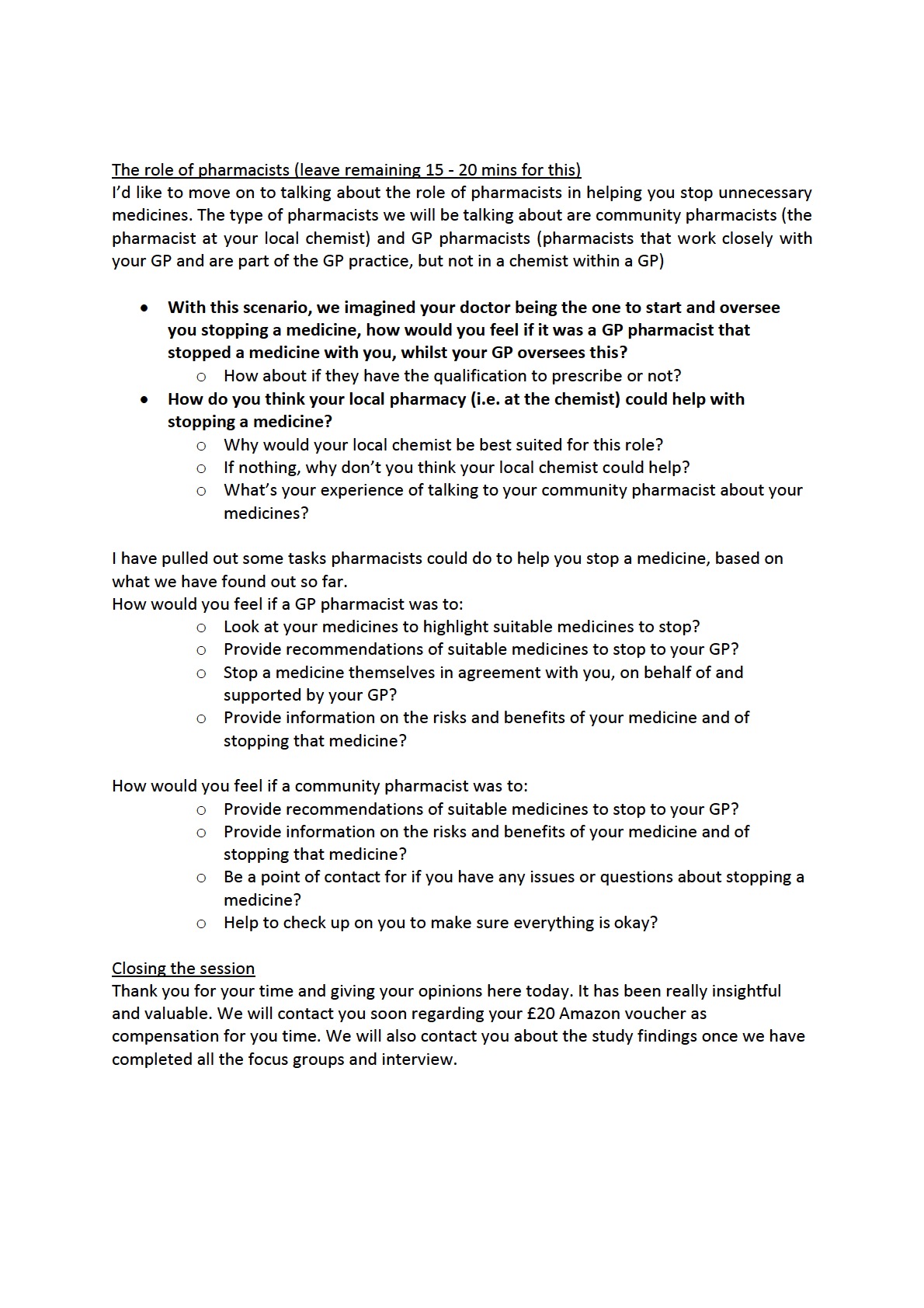


Supplementary file 2: COREQ
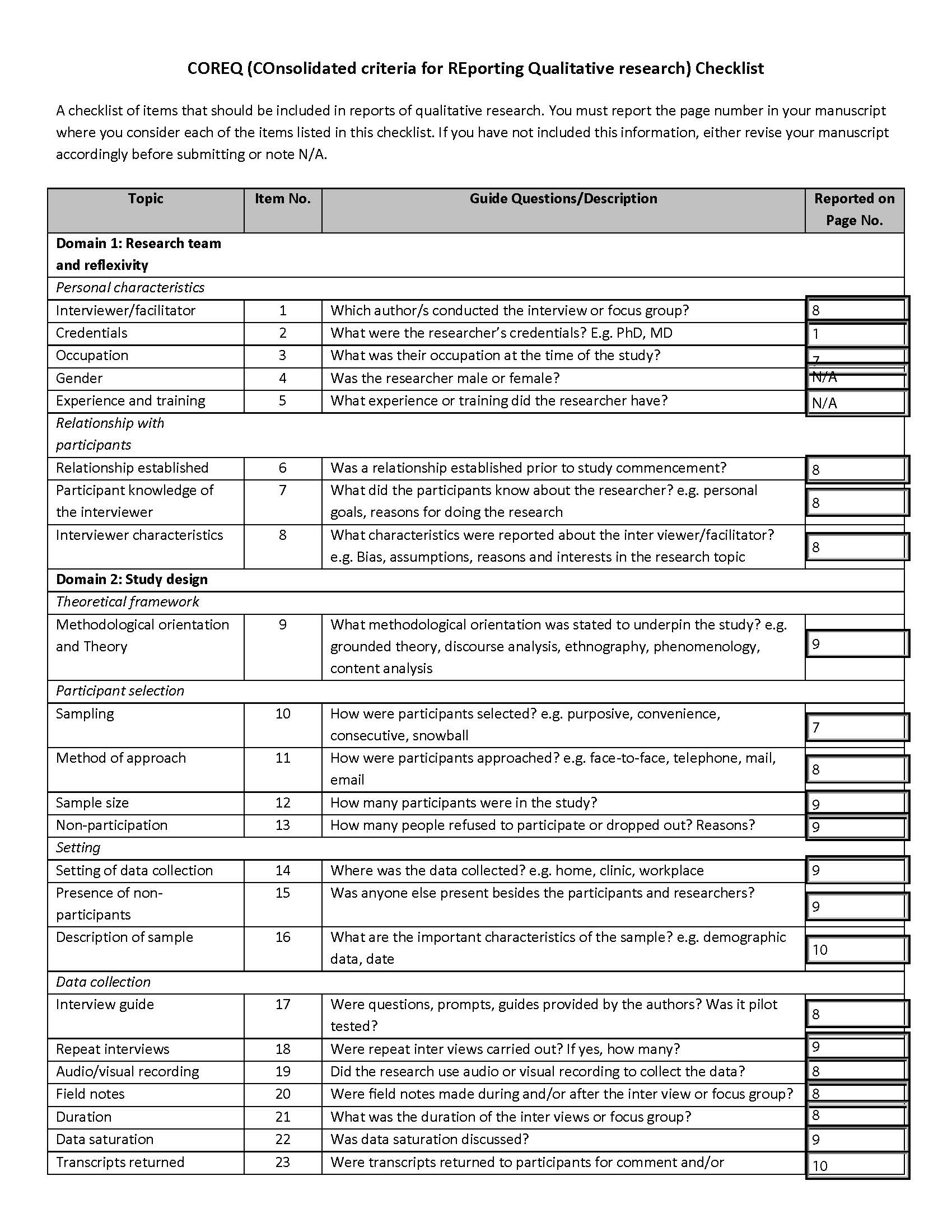

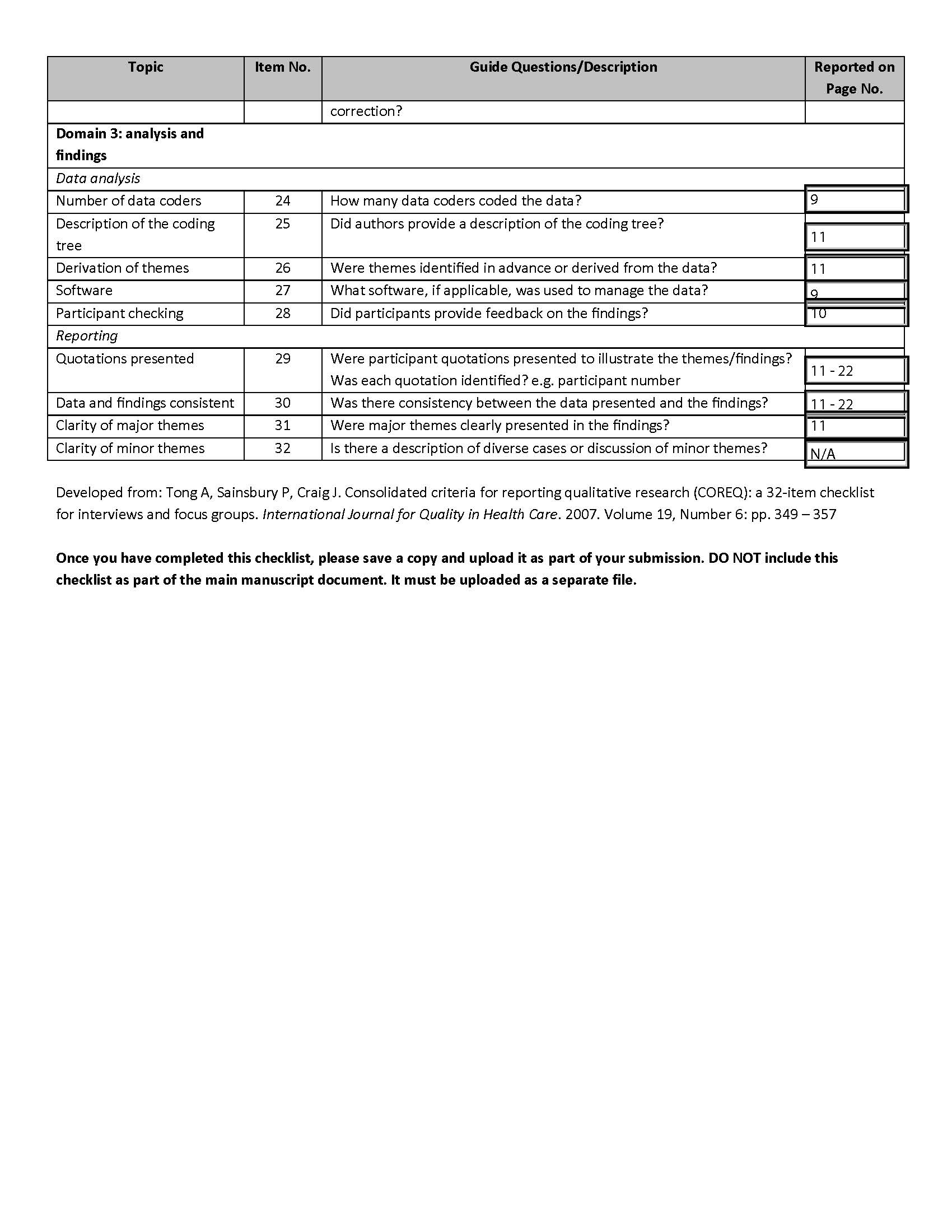

Supplement: Supplementary file 1 — Supplementary Material 1 [file 12877_2024_5435_MOESM1_ESM.docx]
